# Operational strategies, monitoring and control of heterologous protein production in the methylotrophic yeast *Pichia pastoris *under different promoters: A review

**DOI:** 10.1186/1475-2859-5-17

**Published:** 2006-04-06

**Authors:** Oriol Cos, Ramón Ramón, José Luis Montesinos, Francisco Valero

**Affiliations:** 1 Departament d'Enginyeria Química. Escola Tècnica Superior d'Enginyeria (ETSE). Universitat Autònoma de Barcelona (UAB). 08193 Bellaterra (Barcelona), Spain

## Abstract

The methylotrophic yeast *Pichia pastoris *has been widely reported as a suitable expression system for heterologous protein production. The use of different phenotypes under *PAOX *promoter, other alternative promoters, culture medium, and operational strategies with the objective to maximize either yield or productivity of the heterologous protein, but also to obtain a repetitive product batch to batch to get a robust process for the final industrial application have been reported. Medium composition, kinetics growth, fermentation operational strategies from fed-batch to continuous cultures using different phenotypes with the most common *PAOX *promoter and other novel promoters (*GAP, FLD, ICL*), the use of mixed substrates, on-line monitoring of the key fermentation parameters (methanol) and control algorithms applied to the bioprocess are reviewed and discussed in detail.

## Review

### Introduction

A limited number of species belonging to the genera *Hansenula*, *Candida*, *Turolopsis *and *Pichia *are capable of growing on methanol [1-4]. In the early 70's, the interest in the production of single cell protein (SCP) from methanol as the sole carbon and energy source intensified the studies of these specific strains for two reasons: to explore their possible commercial applications and to study the specific cell compartments which are abundantly present in methanol-growth cells, namely peroxisomes [5]. Among them, *P. pastoris *is one of the most cited methylotrophic yeast.

The Phillips Petroleum Company developed the media and fermentation protocols for growing *P. pastoris *in continuous cultures at high cell densities to obtain SCP from methanol [6]. However, production of SCP from this source became highly unattractive because of the high increase in the cost of methanol (Oil crisis of the 1970's).

*P. pastoris *emerged as important yeast in biotechnology one decade later when Phillips Petroleum together with the Salk Institute Biotechnology/Industrial Associates Inc (SIBIA, La Jolla, CA, USA) used this methylotrophic yeast as a system for heterologous protein expression becoming a successful choice both for academic and industrial purposes [7,8]. Nowadays, more than 500 proteins have been cloned and expressed using this system [9,10]. Furthermore, it has been selected by several protein production platforms for structural genomics programs [11-13].

*P. pastoris *combines the ability of growing on minimal medium at very high cell densities, secreting the heterologous protein simplifying their recovery. Also, it performs many of the higher eukaryotic post-traductional modifications such as protein folding, proteolytic processing, disulfide bond formation and glycosilation. But, probably the most important characteristic of *P. pastoris *as host microorganism is the existence of a strong and tightly regulated promoter from the alcohol oxidase 1 gene, *PAOX1 *[14].

Alcohol oxidase is the first enzyme of the methanol assimilation pathway which catalyses the oxidation of methanol to formaldehyde [15]. There are two alcohol oxidase genes in *P. pastoris *that code for the alcohol oxidase enzyme, the alcohol oxidase 1 gene (*AOX1*), which is responsible for greater than 90 of the enzyme in the cell, and the alcohol oxidase 2 (*AOX2*) for less than 10%. There are three types of *P. pastoris *host strains available that vary with regard to their ability to utilize methanol. The wild type or methanol utilization plus phenotype (Mut^+^), and those resulting from deletions in the *AOX1 *gene (methanol utilisation slow Mut^s^) or both *AOX *genes (methanol utilisation minus Mut^-^).

Also a high expression levels using the *PAOX2 *or with a truncated version of the promoter has been reported [16]. However, the expression levels using the *PAOX1 *have been higher than those reported for the *PAOX2*.

In recent years, some efforts have been made to circumvent the use of methanol developing new alternative promoters to *PAOX1*. The glyceraldehide 3-phosphate dehydrogenase promoter (*PGAP*) was isolated by Waterham in 1997 [17] and it has been successfully used for constitutive expression of several heterologous proteins. However, its use is not suitable if the expressed heterologus protein is toxic to the cells. On the other hand, the cloning and characterization of the formaldehyde dehydrogenase gene and promoter has been reported [18]. The *FLD1 *gene codes for an enzyme that plays an important role in the methanol catabolism as carbon source, as well as in the methylated amines metabolism as nitrogen source. Initial characterization studies showed that the *PFLD1 *from *P. pastoris *was strongly and independently induced either by methanol as carbon source or methylamine as nitrogen source [18]. Resina and coworkers have shown that the levels of recombinant *Rhizopus oryzae *lipase were similar among *PAOX1 *using methanol as sole carbon source and inducer [19], and *PFLD1 *using sorbitol as sole carbon source and methylamine as sole nitrogen source and inducer.

In 2003, the production of dextranase from *Penicillium minioluteum *in *P. pastoris *under the control of the novel promoter *ICL1 *(isocitrate lyase) has been reported [20]. However, more studies are necessary to evaluate the performance of this new promoter.

The productivity of a recombinant system depends on several genetic and physiological factors such as the codon usage of the expressed gene, the gene copy number, efficient transcription by using strong promoters, translation signals, translocation determined by the secretion signal peptide, processing and folding in the endoplasmatic reticulum and Golgy and, finally, secretion out of the cell, as well as protein turnovers by proteolysis but also of the optimization of fermentation strategy [21].

However, simply inserting a gene of interest into a vector and transforming a microbial host is no guarantee of a viable process. The expression level for a given recombinant protein produced in *Pichia *seems to be decided largely by its inherent properties such as amino acid sequence, the tertiary structure and the site of expression [22]. As these factors are interdependent, it is not trivial to optimize such a system dependent of several variables.

In this article, an overview about the optimization of operational strategies from medium composition, monitoring, kinetics, operational strategies under different promoters to control process is reported. Obviously, the optimization of other factors would have positive and important effects on the final level of the heterologous product obtained. However, from an industrial point of view, it is important to work not only under the optimal conditions to maximize either yield or productivity of the heterologous protein, but also to obtain a repetitive product from batch to batch under the more controlled and automated possible conditions.

For instance, Boze and coworkers obtained significantly higher levels of expression in a bioreactor [23], compared with the shake flask procedure, when *P. pastoris *was cultivated using a high cell density fed-batch or in continuous process (where the pH and the pO_2 _are controlled). A 30-fold increase in rFSH concentration was observed in bioreactor cultures compared to shake flasks.

### Fermentation media and operational conditions

Bibliographic revisions about the *P. pastoris *production system demonstrate that the best conditions (medium, pH, temperature, etc.) vary according to the kind of strain used and/or the foreign protein expressed [24,25]. Certainly, there are not fixed conditions that ensures the optimal production but there are some guidelines that allow an important improvement of the productivity.

The most common medium for high cell density fermentation of methylotrophic yeast *P. pastoris *is the basalt salt medium (BSM) proposed by Invitrogen Co. [11]. This is considered a standard one, though, it may not be the optimum and may have some important problems (unbalanced composition, precipitates, ionic strength, etc.).

Therefore, alternative media like the proposed by d'Anjou and coworkers [26] or the so-called FM22 formulated by Stratton and coworkers [27] have been described. All of these culture media have been formulated to obtain high cell densities in fed-batch cultures. Comparing the chemical elemental composition of the different culture media important differences can be observed (Table 1). For instance, the variations in the phosphorus and potassium concentrations are significantly important, being higher for BSM and FM22. The BSM medium provides, in general, high concentration of basic elements, all at the upper level of the recommended range proposed by Wegner [28]. This author studied the effect that the main medium elements had on the yeast growth, and defined the best concentration range of each element. Conversely, the d'Anjou medium has a low concentration of chemical elements, around the low limit of Wegner's range. The concentrations of the elements in FM22 media are similar to BSM with the exception of potassium.

**Table 1:** Elemental content comparison between medium compositions. BSM, FM22 and D'Anjou.

**Element**	**BSM (g/L)**	**FM22 (g/L)**	**D'Anjou (g/L)**
N	NH_4_OH (pH control)	1.06 + NH_4_OH (pH control)	4.24
P	12.27	9.76	2.73
K	11.05	18.74	3.45
Mg	1.47	1.15	0.46
Ca	0.27	0.12	0.10
S	5.51	5.46	5.47
Cl	--	--	0.17

One of the most important points in a medium formulation is the nitrogen source. In BSM and FM22 this element is mainly added as ammonium hydroxide when controlling pH. However, in the d'Anjou medium all nitrogen is supplied at the initial formulation and it is not added during the culture.

Cos and coworkers determined the elemental composition of the microorganism in *Rhizopus oryzae *lipase production (CH_1.78_O_0.62_N_0.18_S_0.006_) estimating that nitrogen starvation starts around 50 g/L of biomass [29]. The lack of nitrogen is directly related to the increase in proteolytic activity, and in consequence, to the degradation of extracelular protein [30]. Thus, in BSM and FM22 media, when nitrogen is added to control pH, this effect could be present when high biomass concentration is reached. In some cases, the best simplest solution is its measure and if necessary, to supplement nitrogen. However, it is necessary to avoid its accumulation because it can provoke the inhibition of growth and enlarge the lag phase [31].

All the defined media are supplemented with micronutrients like Fe, Mn, Cu and biotin, normally used in the PTM1 trace salts composition proposed by Invitrogen. The influence of these elements in *P. pastoris *growth has not been widely studied. Only Boze and coworkers demonstrated that addition of seven vitamins and two trace salts in BSM medium improve the production in *P. pastoris *in comparison to the BSM+PTM1 trace salts [23].

In high cell density cultures, foam formation is commonly caused by the high agitation and aeration conditions applied. Generally, mechanical solutions are not enough to reduce its presence and an antifoam agent addition is necessary. The use of silicone based ones is highly efficient but could have negative effects on yeast growth rate and oxygen transfer rates [32].

The optimal growth and production temperature in *P. pastoris *is 30°C, above 32°C protein expression stops and growth quickly decays. However, some authors pointed out that working at low temperatures significantly improves the production of heterologous protein. Li and coworkers decreased the temperature of the culture from 30°C to 23°C and improved 3 fold the production of herring antifreeze protein [33]. Jahic and coworkers showed that using a decay temperature profile during induction phase decreases both, protease activity and cellular lysis [34].

The most commonly pH used in different studies is between 5 and 6, even though *P. pastoris *can grow in a wider range, 3 to 7 [7]. In several cases, the pH value has been fixed around 5.5 to reduce protease effects in the medium [30], and to improve the stability of the foreign protein [34]. The use of BSM medium at pH greater than 5.0 causes precipitation and produces important operational problems like starvation of nutrients and OD measurement interference.

### Growth kinetics

Some authors have reported studies on the growth model from experimental data obtained in methanol non-limited fed-batch and continuous cultures with Mut^+ ^phenotype. It is well documented that a typical growth inhibition profile at high methanol concentration is obtained. Kobayashi and coworkers described this relationship for the production of human serum albumin from data obtained at constant methanol concentration in fed-batch bioprocess, although not growth model was reported [35]. This inhibition profile has been reported by Zhang and coworkers in the production of a heavy-chain fragment C of botulinum neurotoxin serotype A in fed-batch cultures [36], and also in the heterologous hirudin production in fed-batch and continuous cultures [37]. These experimental data have been fit to the following uncompetitive inhibition growth model [38].



Specific growth rate increases with methanol concentration up to a maximum value named theoretical maximum specific growth rate μ^'^_max_, corresponding to a critic substrate concentration (S_crit_)_, _beyond which it declines. Growth characteristics were divided in two regions based upon μ^'^_max _or the equivalent point S_crit_. For methanol concentration values lower than S_crit _is the growth limited region and for values higher than S_crit _is the growth inhibited region. The kinetics values obtained are presented in Table 2.

**Table 2:** Kinetics parameters obtained applying an uncompetitive inhibition growth model.

**Protein**	**μ**_max _**(1/h)**	**k**_S _**(g/L)**	**K**_I _**(g/L)**	**μ**^'^_max_**(1/h)**	**S**_crit _**(g/L)**	**Reference**
Fragment C of Botulinum	0.146	1.5	8.86	0.08	3.65	[36]
hirudin	0.087	1.35	7.08	0.046	3.09	[37]

Other authors have also calculated the specific growth rate as a function of methanol concentration but only in the range of substrate concentration described by Monod equation (growth limited region), for instance, in heterologous production of chymotripsynogen B [39], and *Rhizopus oryzae *lipase [40]. A comparison of the kinetic parameters obtained for the different heterologous proteins is shown in Table 3.

**Table 3:** Kinetics parameters obtained applying a Monod growth model.

**Protein**	**μ**_max _**(1/h)**	**k**_S _**(g/L)**	**S**_crit _**(g/L)**	**Reference**
Fragment C of Botulinum	0.08	~0.4	3.65	[36]
hirudin	0.046	~0.35	3.09	[37]
chymotripsynogen B	0.084± 0.005	0.218± 0.081	4.0	[39]
Human serum albumin	0.16	< 0.2	5.5	[35]
*R. oryzae *lipase	0.059± 0.002	0.223± 0.037	3.5	[29]

From the comparison of the different kinetics values obtained must be pointed out the influence that the different heterologous proteins have on the kinetics growth of *P. pastoris*.

### Operational strategies under PAOX1 promoter

Invitrogen Co, authorized by RCT (Research Corporation Technologies, USA) to develop and sell the *Pichia *expression system for research purposes only provides an operational manual for the fed batch growth of *Pichia *[41] mainly derived from the protocols of Brierley and coworkers [42]. Fed-batch fermentation protocols include three different phases. A glycerol batch phase (GBP), a transition phase (TP) and finally a methanol induction phase (MIP).

### Glycerol batch phase (GBP)

The objective of GBP is to obtain an important level of biomass prior to protein production in the minimum time possible. The maximum specific growth rate of wild type *P. pastoris *growing on glycerol (0.18 1/h) [29] is higher than growing on methanol (0.14 1/h) [42] and this methanol μ_max _is, generally, lower when *Pichia *is producing a heterologous protein because of the negative effect that heterologous protein production has on the microorganism's growth. GBP strategy is independent of the *Pichia *phenotype under *PAOX1 *and, in general, the concentration of glycerol used is about 40 g/L. This concentration is selected because a glycerol concentration over 40 g/L could inhibit growth [41]. Brierley and coworkers recommended a maximum glycerol concentration of 6% [43] and Chiruvolu and coworkers detected levels of ethanol between 0.5 and 2.4% when glycerol concentration was over 7% [44].

The observed biomass to substrate yield (Y_X/S_) (biomass expressed as dry cell weight) was about 0.5. Thus, a final biomass around 20 g/L is obtained at the end of GBP.

The glycerol exhaustion (end of batch phase) was indicated by a sharp increase in the dissolved oxygen (DO). This is the most common parameter used to decide when the transition phase starts.

### Transition phase (TP)

Biomass level must be increased to generate high cell density cultures. This is one of the objectives of the TP jointly with the derepression of *AOX1 *promoter due to the absence of excess glycerol prior to MIP. Some authors selected a constant glycerol feeding rate [45]. Other authors selected an exponential glycerol feeding rate as sole carbon source in the TP to get a growth-limited level. The maximum glycerol specific consumption rate is 0.0688 g/(g h). Some cell growth predictions, from different exponential feeding profiles using this strategy, have been shown by Zhang and coworkers [36]. The final biomass levels reached at the end of the TP depends on the authors but generally is above 30 g/L.

During the TP, glycerol feeding rate is generally complemented with a methanol feeding rate to favour the derepression of *PAOX1*. With mixed feeding, cultures can be primed for methanol induction, which leads to a substantial reduction in the length of this phase, as it has been demonstrated in the heterologous *R. oryzae *lipase production [46]. Also, the supplemented glycerol can strongly support cells to synthesize alcohol oxidase [47]. Different methanol profiles have been reported for the TP. First, increasing methanol feeding rate as a function of response of DO up to a 7.6 mL/(h L) [42]. Second, maintaining methanol concentration at different set-points: 4 g/L [45] and between 1–2 g/L [48]. Third, a starvation phase about 1 hour previous to MIP to assure that glycerol is completely consumed [49]. Fourth, to program a decreased glycerol feeding rate and constant methanol feeding rate [50,51]. From our experience, one of the best TP strategies, independent of *Pichia *phenotype, is a preprogrammed glycerol feeding rate under growth limited conditions and a variable methanol feeding rate to assure a methanol concentration lower than inhibitory concentration (under 4 g/L).

### Methanol induction phase (MIP)

The methanol feeding strategy in MIP, which also dictates the specific growth rate, is one of the most important factors for maximizing heterologous protein production [36,47], since all of the biochemical reactions for protein production are directly or indirectly associated with cell growth [52]. Moreover, MIP may depend on the operational conditions (e.g. temperature, pH, and culture medium), phenotype and specific characteristics of the heterologous protein produced. The synthesis, processing and secretion of the protein could also affect cell growth. Different approaches have been proposed to optimize MIP. These strategies are commented in the control schemes section.

### Methanol monitoring

One of the most important key parameters in *P. pastoris *expression system is the methanol concentration. Monitoring and controlling this variable are important because high levels of this inductor substrate can be toxic to the cells [47] and low levels of methanol may not be enough to initiate the AOX transcription [6]. Keeping a constant methanol concentration during the induction phase has positive effects on the production of foreign protein [53].

In last years, large number of equipment to monitoring methanol in *P. pastoris *cultures has been developed. The main off-line techniques are gas chromatography, HPLC and enzymatic reactions, used generally in a batch or fixed pre-programmed methanol addition techniques. More advanced monitoring off-line techniques have the ability to monitor multiple compounds of media in addition to methanol. A Near Infrared spectroscopy (NIRS) technique was used to model key analytes (biomass, glycerol, methanol and product concentration) in the production of a therapeutic mammalian protein by *P. pastoris *in high cell density culture [54]. These methods require a pre-treatment of the sample and its time response can be so long for control purposes.

Some on-line techniques are based on automates, the most typical off-line techniques to achieve an autonomous measure system. The Sequential Injection Analysis (SIA) developed by Surribas and coworkers is an example, capable to measure seven samples per hour with low standard deviation (RSD: 4%) [55]. Also commercial equipment with similar characteristics and ready to connect to the bioreactor such as the YSI 2700 select analyzer (Yellow Springs Instruments, Ohio, USA) can be found. However, continuous extraction of samples increases contamination probability besides drop of volume may be significant.

Based on the gas liquid phase equilibrium, there are two different techniques for using an organic vapour sensor (TGS822, Figaro electronic Co. Ltd., Tajin, China) to methanol monitoring in fermentation broth. First, detecting methanol vapour that permeates from the culture broth across a silicone tube [56,57], or by using a probe inserted into the bioreactor [58,59]. Second, directly detecting the methanol vapour in the stream gas outlet [36,39,51]. This last strategy is the most commonly used for the monitoring and control of the methanol concentration and different experimental set-up has been proposed [60]. Equipment based on this sensor has been commercialized in an exhaust gas flow of bioreactor (MC-168, PTI instruments Inc., Kathleen, USA) or directly in a culture media (ALKOSENS, Frings America, Illinois, USA). Presence of other volatile organic vapour and ammonia derived compounds in the gas phase can interfere, although this can be solved with simple system modifications [60].

### Control schemes

When the objective of the research is, for instance, to obtain small quantities of heterologous protein for structural studies, the methanol feeding strategy from "*Pichia *fermentation process guidelines" of Invitrogen Co. (San Diego CA) using shaken-flasks cultures under non-controlled conditions could be satisfactory [61]. When the objective is to maximize product yield, the selection of the optimal methanol feeding strategy in the induction phase, and the production in a bioreactor under controlled conditions is necessary, also in order to achieve a high and reproducible product quality.

When on-line methanol concentration monitoring is not available two different methanol feeding strategies are the most commonly used [27]: The so called DO spike method, and the implementation of an open-loop methanol control strategy.

In the first method, methanol feeding rate is modified in order to avoid methanol exhaustion as indicated by a sharp increase in dissolved oxygen. Some authors have reported this strategy [62,63].

Rodríguez-Jiménez and coworkers tested three different strategies to control the methanol flow rate in cultures of a dextranase-producing Mut^s ^clone of the methylotrophic yeast *P. pastoris *[63]. The increment of methanol-feeding rate used in the strategy based on dissolved oxygen (DO) in the culture medium gave better results for both cell growth and enzyme production than using manual control of the methanol feeding rate, reaching 56.2 g of biomass and 5.14 g of dextranase per litre.

Lim and coworkers reported a DO-stat control strategy for two variables in the rGuamerin production process in a repeated fed-batch culture [62]. The ratio of partial pressure of pure O_2 _in the inlet air-stream and the methanol feed rate were controlled simultaneously. By using this control strategy, methanol feeding in the induction phase could be controlled automatically while efficiently controlling the dissolved oxygen level. As a result, the cell concentration reached more than 140 g l^-1 ^and rGuamerin expression reached a level that was 40% higher than achieved in a fed-batch process using manual control of the methanol feeding rate.In order to improve the performance of such DO-based methods, schemes based on feedback control strategies have been developed (DOstat). In these bioreactor feed controllers, the methanol feeding rate is adjusted in a manner determined by a control algorithm which attempts to control the level of dissolved oxygen in the reactor. Oliveira employed an adaptive dissolved oxygen control [64]. Chung designed a feedback controller based on the Bode stability criterion [65]. However, despite some authors used this DO stat method, to increase process robustness and/or process performance neither methanol concentration nor specific growth rate were constant within this strategy. This fact makes very difficult to study the influence of these variables on the production of the heterologous protein.

DO spike method has an additional problem, if an inhibitory methanol level is reached, a sharp increase in dissolved oxygen will be observed, the response of the system will be then to increase the methanol feeding rate and subsequently, a higher methanol accumulation in the bioreactor will take place. In consequence, methanol concentration not longer be optimal for heterologous protein production.

The method based on the methanol control using an open-loop structure, a feeding rate profile derived from mass balance equations to theoretically maintain a constant specific growth rate (μ) was implemented. This approach is based on simple cell growth models with no on-line information about the system. To implement this method, a constant biomass to substrate yield (Y_X/S_) for the fed-batch induction phase is considered and, biomass concentration and volume are supposed to be known at the beginning of the preprogrammed feeding strategy.

Zhang and coworkers, empirically developed a methanol feeding strategy based on a growth model, obtaining μ when performing Invitrogen's feeding protocol, to maximize the protein production [36]. D'Anjou and Daugulis, using an exponential feeding strategy with mixed glycerol/methanol substrate in a fed-batch, demonstrated the usefulness of a rational, model-based approach for improving the productivity of recombinant *P. pastoris *fermentation [48]. Trinh and coworkers, evaluated three different strategies, one responding to the methanol consumption using a methanol sensor, the second responding to the oxygen consumption and the last based on a predetermined exponential feeding rate, controlling the growth rate at 0.02 (1/h), in this last strategy the methanol supply is limited. Total protein production was similar in all three strategies (133 mg/L) but the amount of methanol added and the biomass produced were lower in the predetermined rate method. This caused that the specific production of endostatin was 2 fold higher in the predetermined rate than in the other two methods [57]. Sinha producing recombinant ovine interferon-τ (r-oIFN-τ), modelled cell growth on methanol using a substrate-feed equation, which served as the basis for the open loop control. The optimum conditions for the protein production was obtained maintaining μ at 0.025 (1/h) [24].

In the two general approaches described above, the methanol concentration is neither on-line determined nor directly controlled and so, significant deviations from optimal methanol concentration and even periods of exhaustion and build-up can be observed. Methanol concentrations during the induction phase directly affect cellular growth and protein yield. Thus, an accurate monitoring and control of the methanol concentration is required if a robust and reproducible bioprocess is wanted. Different analytical approaches have been applied to monitor methanol concentration during *P. pastoris *fermentation, all described before.

Although open loop systems could be easy to implement they have no response to possible perturbations of the system. Simplest closed loop control strategy is the "on-off" control mode. Some works have been published with apparently satisfactory results [36,51,56,66]. However, bioprocess in general, and *P. pastoris *heterologous protein production in particular, are characterized by a complex and a highly nonlinear process dynamics. In this way, a simple "on-off" control strategy is inadequate for precise control of methanol concentration in the bioreactor because it can result in a fluctuating methanol concentration around the set-point.

A PI or PID control algorithm can be employed to improve the control of the methanol concentration in the culture broth. Nevertheless, the optimal settings of the PID controller are hardly ascertained by trial and error tuning or other empirical methods. Some authors developed a PID control Bode stability criterion to achieve the parameters associated to this kind of control, obtaining good results on methanol regulation in short time fermentations [67].

Because of the dynamics of the system, the optimal control parameters may vary significantly during the fermentation. Moreover, the existence of an important response time for both the on-line methanol determination and the biological system has promoted the development of other control alternatives.

In this way, model-based on-line parameter estimation and on-line optimization algorithms have been developed to determine optimal inducer feeding rates to maximize productivity in high-cell-density *E. coli *cultures [68]. Kobayashi and coworkers investigated the optimal specific growth rate with the method of dynamic programming, where a mass balance model was used [35]. Continuous fermentation using methanol was performed via on-line methanol measurement and control using a minimal-variance-controller and a semi-continuous Kalman-Filter [45].

### Fed-batch fermentation

It is very difficult to compare the performance between different fed-batch strategies with different heterologous proteins. Establishing optimal protein expression protocols should therefore include the evaluation of various growth and induction strategies in addition to other environmental parameters.

Different strategies have been implemented in the production of *Rhizopus oryzae *lipase in *P. pastoris *Mut^+ ^phenotype. A methanol feeding fed-batch strategy under off-line methanol concentration control including a transition phase led to 20–30 h reduction in the production time, a 11-fold higher final lipolytic activity, a 13.6-fold higher productivity and a 10.3-fold higher specific productivity compared to the DO-based strategy [46].

A different growth rate could result in a different production rate in fed-batch fermentation. Zhang and coworkers proposed a rational feeding strategy that can deliver a constant desired μ for a limited growth for the optimization of the production of BoNT-A-(Hc) [36]. This feeding strategy applies an exponential feeding rate to result in a desired μ of 0.0267 (1/h) for maximum production.

Trinh and coworkers compared two strategies, one based on the metabolic activity of the yeast and a second strategy based on supplying methanol at a predetermined exponential rate (limited methanol supply; constant μ = 0.02 1/h) in the heterologous production of mouse endostatin with a Mut^+ ^phenotype. The last strategy was more efficient than the other two.

The higher efficiency of methanol utilization at the predetermined exponential rate suggests that the methanol is directed mainly towards energy generation, and that only a small portion is directed to biomass production [57]. The results are in good agreement to the evidence that when cells are fed with methanol at a growth-limiting rate, the *AOX1 *is induced to levels from 3 to 5 times higher than in cells growing in excess of methanol [69].

Kupcsulik and Sevella [70] showed that neither pH nor temperature has significant effect on the specific growth rate in the range of HSA formation in Mut^s ^phenotype. This implies that the volumetric productivity is mainly determined by the specific product formation rate. This observation supports the idea that, although the capability of cells to grow extends for more acidic pH and higher temperature range, the product formation phase of the fermentation should be conducted at more limited conditions.

### Mixed substrates

It is well known, that one approach to increase the productivity of *P. pastoris *phenotypes is the use of a multicarbon substrate in addition to methanol. It is a simple strategy to increase the energy supply to recombinant cells and the concentration of the carbon sources in the culture broth [47,51,71]. A mixed feed reduces the induction time, increases cell density and volumetric productivity [25].

The mixed feeding strategy is generally employed for Mut^s ^*Pichia *fermentations because of the slow methanol utilization which requires large induction times (above 100 h), although it has also been used for Mut^+ ^strains [72].

Glycerol has been the most feeding co-substrate used at limited rates, ensuring good cell growth while inducing the expression of the heterologous protein [73]. However, with the volumetric productivity enhanced, the specific productivity of recombinant protein may be lower for excess glycerol represses the *AOX1 *promoter [74].

First attempts using glycerol were made by Brierley and coworkers [42] and Loewen and coworkers [75]. While this approach leads to increased overall productivity, the maximum level of protein expression was not reached due to the partial repression of the AOX1 promoter by even slight levels of residual glycerol. McGrew and coworkers doubled growth and CD40 ligand expression levels compared to feeding methanol alone employing mixed feed at a 1:1 methanol:glycerol ratio [76].

Chiruvolu and coworkers showed that higher feed rates of glycerol resulted in increased cell mass production, and decreased specific activity by ethanol accumulation when using a defective Mut^- ^strain [53]. The lowest glycerol feed rate tested produced maximal protein. However, it seemed that extended periods of glycerol-limited feed rates caused sufficient imbalance in energy or amino acids pools in the cell and resulted in the breakdown of a proportion of recombinant β-galactosidase in cells.

In a glycerol-methanol mixed feed fermentation strategy, attention must be paid to determining the appropriate ratio of methanol to glycerol in the substrate feeding rate [73].

Working with Mut^s ^phenotypes Files and coworkers showed that the addition of glycerol during the induction phase increased the concentration and productivity of cystatin C in the early stages of induction in a fed-batch production with a constant methanol feeding rate of 1.8 g/L h and different glycerol feeding rates from 0 to 3.5 MeOH-Gly (v/v). However, the concentration of heterologous protein reached a maximum, levelled off and, when high glycerol feed rates were used, decreased. Although productivity increased up to 1.6 times at low relations MeOH:Gly, maximum concentration of cystatin was not as high as the heterologous protein produced after 96 h induction on methanol [25].

It has also been reported that there is an optimal maximal specific growth rate during *P. pastoris *methanol fed-batch culture, which, when exceeded represses heterologous protein production. Adding methanol at the optimal feeding rate and adding glycerol at a rate that is 20% of the rate for maximal growth in the presence of glycerol increased heterologous protein production by 50% [71].

Interesting papers have also reported the use of glycerol as mixed substrate in *P. pastoris *Mut^+ ^phenotype. Katakura and coworkers showed that the specific growth rate in the presence of glycerol feeding was about 20% higher than that in absence of glycerol feeding in the production of human β_2_-glycoprotein *I *domain V in fed-batch process at a constant methanol concentration of 5.5 g/L. The specific production rate was also 2.3 times higher. These results demonstrate that the glycerol feeding increase the energy supply and the productivity of foreign proteins in *P. pastoris *[51].

Hellwig and coworkers tested different constant glycerol feeding rates maintaining constant the methanol concentration at 5 g/L in the bioreactor. The highest level of heterologous protein, obtained at the lowest glycerol feeding rate tested, was twice lower than fermentation without glycerol addition. Probably, these unexpected results could be due to the glycerol accumulation along the fermentation, especially at the beginning of the fermentation, repressing the expression of the single-chain antibody. However, this data was not showed [58].

Zhang and coworkers, in a complete study, analyzed the influence of mixed-feeds of glycerol and methanol to produce the heavy-chain fragment C of botulinum neurotoxin serotype C in a *P. pastoris *Mut^+ ^phenotype. They designed a fed-batch strategy at an optimal growth rate for maximal production (μ = 0.015 1/h) on methanol feed alone and a preprogrammed feeding strategy with glycerol growth rate ratios ranging from 1 to 4. They concluded that during growth on a mixed feed with μ_Gly _≤ 0.06 (1/h) for this strain, the supplementary feeding of glycerol enhanced the overall growth rather than functioned as a repressor. This observation indicates that it is feasible to use a mixed feed in Mut^+ ^*Pichia *fermentations without growth inhibition by glycerol when the feeding strategy is properly designed [71].

Kuwae and coworkers found that the presence of formaldehyde in the culture supernatant could inactivate the heparin cofactor activity of recombinant human antithrombin. The implementation of a mixed feed process (glycerol-methanol 99:1) resulted in a 40% reduction of the final rAT concentration compared with methanol feed process. However, the specific HC activity was higher prevent partially the negative effect of formaldehyde accumulation [77].

The mixed strategy has also been used to decrease immunotoxin proteolysis and to enhance immunotoxin production [78].

D'Anjou and Daugulis compared the performance of CSTR and fed-batch using glycerol and methanol as mixed substrates to improve the heterologous production of sea raven AFP [48]. In a continuous strategy, the maximum production was achieved at a low dilution rate. However, the maximum specific productivity was reached at the highest dilution rate. Due to glycerol's repression of foreign protein production, the average specific growth rate of the fed-batch culture must be kept sufficiently far from μ _max _to ensure that no residual glycerol is allowed to accumulate in the broth. Therefore, the authors propose to work at μ < 1/2 μ_max_[48].

Although glycerol appeared to be a growth-inhibitory substrate at high concentrations, it increases the rate of extracellular protein accumulation, as it has been shown in the production of human serum albumin in a cyclic fed-batch culture (CFBC). The application of this new fermentation strategy in *P. pastoris *is at least as productive as classical methods with the advantage that it is technically simpler than fed-batch or chemostat cultures [79]. Thus, one of the challenges in mixed substrate strategies is to substitute glycerol for other co-substrate, which not repress AOX promoter.

In *Hansenula polymorpha*, alcohol oxidase (MOX) is derepressed during the exponential growth on carbon sources such as sorbitol, glycerol, ribose and xylose [80]. Although Hansenula *polymorpha MOX *gene and *P. pastoris *AOX genes are not regulated identically, they show common futures of their expression patterns [81]. Although *AOX1 *gene is not fully derepressed in any limited or unlimited carbon sources (> C_1_-carbon chain), non-limiting glycerol and carbon starvation cause some degree of derepression of the promoter [82].

Inan and Meagher compared different carbon sources in terms of their ability to support growth and expression of an *AOX1-lacZ *fusion in shake flasks studies of a *P. pastoris *Mut^- ^strain [81]. It was very clear that glucose, glycerol, ethanol and acetate supported growth but repressed the expression of β-gal. On the contrary, growing on media containing alanine, mannitol, sorbitol and threhalose expressed as much or higher amount of β-galactosidase compared to the Mut^+ ^strain. Recently, lactic acid has also been referred as non-repressing substrate [74].

The use of a less repressing carbon source may result in higher specific production rates, improving overall productivity and eliminating the need for such tight control of residual substrate levels [73]. Among them, sorbitol is a widely accepted non-repressive carbon source for *P. pastoris*.

Thorpe and coworkers compared methanol/glycerol and methanol/sorbitol fedbatch mixed-feed strategies maintaining the residual methanol concentration between 1–2 g/L [73]. Although cell yields are lower on sorbitol, this is compensated by higher specific product formation rates, which results in comparable recombinant protein levels at lower final cell concentrations. The use of sorbitol largely eliminates the concern of ensuring that none of this carbon source accumulates in the medium (unlike the case for glycerol). This implies that sorbitol can be fed at non-limiting rates without the fear of repressing the *AOX1 *promoter, and this makes the process less constrained by the need for tight control of substrate levels.

The use of sorbitol + methanol mixed feed batch fermentation to accomplish several cycles of MMP-2 production was successfully implemented. The approach reduces overall methanol consumption, as most of the growth is supported by sorbitol; and methanol can be added to the sorbitol feed at any desired point to initiate induction of expression [22].

Boze and coworkers showed that rFSH concentration and yield on methanol/glycerol were only half the value observed on pure methanol, when working with the production of rFSH in fed-batch cultures, although biomass was 1.3 times higher. However, when they used sorbitol jointly with methanol as mixed substrate and the medium was enriched with vitamins and yeast extract the production was four times higher than when growing on methanol and the productivity and specific productivity was 4 and 2 times higher respectively. Sorbitol was therefore more suitable to support cell growth and improved rFSH concentration.

When the continuous culture using a methanol/sorbitol medium was the selected strategy, the best results in terms of rFSH production and productivity were reached at the lowest dilution rate tested. These results were also better than those obtained in fed-batch fermentation. The authors showed that while rFSH synthesis is inhibited by glycerol, it is stimulated by sorbitol feeding [23].

Xie and coworkers made an interesting comparison of the effect of different carbon sources on the production of angiostatin with a Mut^s ^phenotype. The strategy used in the induction phase was to maintain a constant methanol concentration of 5 g/L and using DO concentration as indicator to avoid over feeding of glycerol, or a preprogrammed exponential feeding rate when sorbitol was used. They suggested that lactic acid is a potential non-repressive carbon source for expression of foreign genes in *P. pastoris *because the highest angiostatin production level was reached with this substrate, even with lactic acid accumulation up to 6.3 g/L along the fermentation [74].

A summary of different strategies and conditions, productions, productivities and specific productivities is presented in Table 4.

**Table 4:** Mixed substrates cultures comparison

**protein**	**phenotype**	**co-substrate**	**feeding strategy**		**μ**	**production**	**productivity**	**specific productivity**	**Reference**
					1/h (gDCW/L h)*	mg/L (μmol/L)*	mg/L h (μmol/L h)*	mg/gh (μmol/g DCW h)*	

immunotoxin A-dmDT390-bisFv(G4S)	Mut^+^	glycerol	Methanol:glycerol 4:1		----	37	0.55	---	[78]

mAb4813	Mut^+^	glycerol	Methanol constant at	constant glycerol feeding rate at 4.9 g/L h	0.023	~0	---	---	[58]
				
			5 g/L	constant glycerol feeding rate at 2.46 g/L h	0.016	~0	---	---	
				
				constant glycerol feeding rate at 1.23 g/L h	0.007	25	0.28		
				
				No glycerol	0.012	45	0.52		

angiostatin	Mut^s^	glycerol	methanol controlled at 5 g/l glycerol function of OD		0.012	108	1.66	0.019	[74]
			
		sorbitol	methanol controlled at 0.5 g/l sorbitol pre-programmed at constant μ.		0.018	141	2.76	0.030	
			
		lactic acid	methanol controlled at 0.5 g/l lactic acid pre-programmed		0.011	191	2.96	0.044	

cystatin-C	Mut^s^	glycerol	constant methanol feeding rate 1.8 g/L h	constant glycerol feeding rate at 2.1 g/L h	0.048*	45*	0.6*	0.008*	[25]
				
				constant glycerol feeding rate at 3.5 g/L h	0.81*	34*	0.96*	0.009*	
				
				constant glycerol feeding rate at 6.4 g/L h	1.38*	15*	0.95*	0.008*	
				
				No glycerol	0.08*	54*	0.6*	0.011*	

sea raven AFP	Mut^s^	glycerol	continuous		0.01	60	0.6	0.015	[48]
					0.09	15	1.6	0.065	
				
			fed-batch a constant μ = 0.03 1/h		0.03	180	2.3	0.04	
				
			fed-batch a constant μ = 0.07 1/h		0.07	120	2.4	0.04	

rFSH	Mut^s^	only methanol	pre-programming feeding fed-batch			45	0.3	0.014	[23]
					
		glycerol				27	0.2	0.011	
					
		sorbitol				187	1.3	0.035	
		only methanol	continuos		0.012	170	2	0.033	
					
		sorbitol			0.005	282	1.4	0.018	
					
		sorbitol			0.01	121	1.1	0.018	
					

sea raven AFP	Mut^s^	glycerol	Methanol	fed-batch		180	1.5	0.045	[73]
					
		sorbitol	constant at 1–2 g/L	fed-batch		200	1.7	0.060	

β-galactosidase	Mut^-^	glycerol	Methanol constant at	constant glycerol feeding rate at 1 g/L h		415 U/mL	8.5 U/g h	7865 U/g h	[53]
						
				constant glycerol feeding rate at 4 g/L h	568 U/mL	10.7 U/mL h	3717 U/g h		
						
				constant glycerol feeding rate at 7 g/L h	577 U/mL	12.3 U/mL h	3005 U/g h		
						
				step increase glycerol feeding rate	340 U/mL	6.7 U/mL h	4392 U/g h		

### Continuous fermentation

Traditionally *P. pastoris *fermentations are performed in fed-batch mode using the methanol-inducible system, because fermentation technology for *P. pastoris *is still based on the SCP process. Thus, high-cell-density fed-batch fermentation is mainly applied for the production of recombinant proteins [39].

However continuous production mode offers, in comparison to fed-batch fermentation, advantages in terms of higher volumetric productivity, product quality, product uniformity and the exposure of the product to the proteolytic enzymes, meanwhile oxidation or inactivation is significantly reduced [83]. Continuous operation provides a greatly enhanced production of recombinant proteins (approximately five to six fold higher productivity than fed-batch fermentation) and a reduction of downtime associated with fermentation turnaround [84]. Thus, in the last years continuous strategy appears as an alternative to high-cell-density fed-batch strategy.

Continuous operation using methanol as sole carbon source under control of *PAOX1 *is practically limited to Mut^+ ^phenotype. The low maximum specific growth rate of Mut^s ^phenotype limits the operational dilution rate and thus, the productivity of the process.

However, Boze and coworkers producing a porcine follicle-stimulating hormone (rFSH) in a continuous process with a Mut^s ^clone obtained, at a dilution rate of 0.012 (1/h), a rFSH concentration and Y_X/S _3.7 and 2.3 times higher than in fed-batch cultures with a productivity (mg rFSH h^-1^) and specific productivity (q_p_;mg rFSH/g × h) 6.4 and 2.3 times higher [23].

As we have reported in the mixed substrate chapter, one approach to increase the productivity of Mut^s ^phenotype is the use of a mixed carbon feed. Glycerol and sorbitol are the most commonly used co-substrates jointly with methanol. With both substrates, but specially with glycerol, where the repression effect of foreign protein production is well known, the continuous strategy is to select a dilution rate far enough from μ_max _to ensure that no glycerol is allowed to accumulate in the broth, and also methanol concentrations have to be maintained at levels sufficient to fully induced heterologous protein production, yet no so high as to be inhibitory to cell growth or heterologous protein expression [48].

D'Anjou and Daugulis performed a set of CSTR experiments to determine the relationship between the dilution rate (in a range between 0.01 (1/h) and 0.09 (1/h)), the specific methanol consumption rate, and the specific production rate in the heterologous production of a sea raven antifreeze protein using mixed methanol/glycerol feed [85]. The specific methanol consumption rate as a function of the dilution rate showed a maximum at approximately D = 0.05 1/h. Maximum production was achieved at the lowest dilution rate. However, the maximum specific production rate (q_p_) was achieved at the highest dilution rate. This behaviour confirms that product formation is related to growth by a constant yield coefficient. Finally, they demonstrated that a CSTR system will yield a higher productivity compared with a fed-batch system.

Boze and coworkers found that using sorbitol and methanol as mixed substrates in continuous fermentations do not increase the protein production, productivity and specific productivity compared with continuous fermentations using methanol as sole carbon source at the same dilution rate (0.01 1/h). Although protein production was 1.65 fold higher using mixed substrates when dilution rate was reduced at 0.005 (1/h), both, productivity and specific productivity were lower than continuous using methanol as carbon source. A more precise control of the sorbitol concentration must be necessary because the authors detected variation of residual sorbitol concentration from 4 to 14 g/L [23].

First continuous fermentations with Mut^+ ^phenotype, using methanol as carbon source, were made for the extracellular production of bovine lysozyme c2 [42,86]. Similar to Mut^s^phenotype performance, the highest production was reached at a lower dilution rate (0.035 1/h). However, in terms of productivity, intermediate dilution rates are probably the best option. Productivity of CSTR was 2 times higher than that of standard fed batch fermentation.

Curvers and coworkers comparing heterologous production of chymotripsinogen B in fed-batch and CSTR fermentations showed that the productivity of CSTR was 4.7 fold higher at a dilution rate of (0.072 1/h). The highest productivity in CSTR could also be related to the cell age distribution in fed-batch process and to the prolonged production phase in continuous cultures, no product is formed during one-third of the entire fed-batch process [45].

Heterologous protein production is considered to be mainly growth-coupled. Thus the increased growth rate during continuous fermentation inevitably will give rise to higher productivity. This affirmation could be correct for the productivity but not for protein production, where several authors have demonstrated that the maximum production is achieved at low dilution rates [42,87].

Beyond the possible improvement of productivity, also serves as a tool for determination of growth and product formation kinetics and it has used in the production of chymotripsinogen B [39]. For instance, former analysis of growth kinetics has been limited to quasi-steady-state data. However some parameters obtained from continuous culture, in special the parameters related to protein production, not always can be used for fed-batch processes [87].

Curvers and coworkers detected that when methanol concentration exceed a critical value, it imposes a selective pressure against product formation leading to the enrichment of non-producing mutants. These mutants exhibit strongly increased substrate consumption and growth rates and outcompete the slow-growing production strain in continuous fermentation. Even significantly lower methanol set points often resulted in phenotypic destabilization indicating that a transient overshoot is sufficient to trigger this phenomenon. To thoroughly understand the destabilization of production strains during continuous fermentation, genetic analysis of the strains reisolated from the experiments will be necessary [39].

A robust study to maximize the productivity of interferon τ (INF-τ) optimizing biomass concentration and dilution rate using response surface methodology (RSM) was made by Zhang and coworkers [87]. They found that the relationship between μ and specific methanol consumption rate (q_S_) is about the same as that obtained from fed-batch fermentations from the same strain and also quite similar to other *P. pastoris *strains [47,71,72]. However, in terms of heterologous protein production parameters, the optimal conditions for maximizing specific productivity and productivity may not be necessarily at the same dilution rate. The authors have also noted that the optimal μ (D) for the maximum q_p _obtained in CSTR (0.033 1/h) was different from that (0.025 1/h) obtained in fed-batch fermentations.

More recently, a constitutive promoter *GAP *is being used as an alternative to *AOX *promoter. However, in some heterologus protein production with negative effect on specific growth rate this promoter is inefficient.

Goodrick and coworkers described the first report of *P. pastoris *high-cell-density fermentation where h-chitinase was produced in continuous culture for 30 days. The system provided not only for greatly enhanced production (five to six folds higher than fed-batch fermentation) but also the production of intact proteins that are usually degraded in fed-batch fermentation. This may be due to the continual separation of sensitive proteins from the culture broth and/or a reduction of level of protease(s) in the culture [84].

Table 5 presents a summary of the more relevant results obtained in continuous fermentation.

**Table 5:** Continuous cultures comparison.

**Protein**	**Promoter**	**Operation**	**Mixed substrates**	**D (1/h)**	**Protein (mg/L)**	**Productivity (mg/L h)**	**Y**_P/X _**(mg prot/g X)**	**Specific productivity (mg prot/gX h)**	**Reference**
porcine FSH	*AOX *Mut^s^	Fed-batch	methanol	--	45	0.3	1.2	0.014	[23]
			gly-met	--	27	0.2	0.6	0.011	
			sor-met	--	93	0.6	0.9	0.016	
			sor-met-cas	--	72	0.5	1.4	0.017	
			sor-met-ye	--	113	0.8	1.5	0.019	
			sor-met-v-ts	--	187	1.3	2.5	0.035	
			
		Continuous	methanol	0.012	170	2.0	2.8	0.033	
			met-sor-v-ts	0.005	282	1.4	3.6	0.018	
				0.01	121	1.1	1.8	0.018	

Sea raven antifreeze	*AOX *Mut^s^	Fed-batch	gly-met	μ = 0.03	180	2.3	3	0.038	[48]
protein				μ = 0.07	120	2.4	2	0.040	
					
		Continuous		0.01	60	0.6	1.5	0.015	
				0.02	40	0.8	1	0.020	
				0.03	50	1.5	1.3	0.040	
				0.05	15	0.8	0.5	0.025	
				0.07	15	1.1	0.7	0.050	
				0.08	15	1.2	0.8	0.060	
				0.09	15	1.4	0.7	0.065	

Bovine lisozyme	*AOX *Mut^s^	Fed-batch	methanol	--	250	1.2	5.2	0.026	[42]
			gly-met 4:1	--	180	3.4	2.3	0.046	
			gly-met 2:1	--	290	4.8	3.7	0.062	
			Non-limiting methanol	--	375	5.6	4	0.093	
		
	*AOX *Mut^+^	Fed-batch	methanol	--	450	7.7	5.6	0.154	
		Continuous	methanol	0.035	600	12–15	--	--	

Chymotripsynogen B	*AOX *Mut^+^	Fed-batch	methanol	--	475	5.3	3.2	0.035	[39,45]
			
		Continuous	methanol	0.038	368	14	--	--	
				0.062	339	21	--	--	
				0.072	347	25	4.3	0.31	

Ovine interferon-ô	*AOX *Mut^+^	Continuous	methanol	0.008	45	0.4	0.9	0.007	[87]
				0.0136	149	2.0	2.3	0.031	
				0.035	85	3.0	1.3	0.047	
				0.05	39	2.0	0.5	0.027	

h-chitinase	*GAP*	Fed-batch	glucose	--	300	2.5	7.5	0063	[84]
				--	450	2.7	6.8	0.040	
					
		Continuous		--	300	15	2.1	0.150	

h-chitinase	*GAP*	Continuous	glucose	0.042	250	6.1	2.8	0.067	[83]

gly = Glycerol; met = Methanol; sor = Sorbitol; cas = casaminoacids; ye = yeast extract; v = vitamins; ts = trace salts.

Other more complex continuous schemes than chemostats and nutriostats have also been reported. Perfusion culture technique using a shaken ceramic membrane flask (SCM flask) has been applied to the recombinant production of β-galactosidase [88,89]. A rotary membrane separation system was used in a continuous fermentation with cell recycling to obtain high cell concentration and high thrombomodulin production [49].

The question of optimal operating mode and operating conditions has to be settled on issues beyond the volumetric productivity. Product concentration and the concentration of other proteins in the fermentation culture play a key role in determining the downstream processing steps, and the entire process needs to be evaluated on an overall cost basis, where issues such as oxygen supplementation, cooling, and raw materials costs need to be considered in the context of overall process [48].

### Alternative promoters

In recent years, some efforts have been made to circumvent the use of methanol. Alternative promoters such as the glyceraldehide 3-phosphate dehydrogenase promoter (*PGAP*) and the formaldehyde dehydrogenase promoter *(PFLD1) *have been developed [6].

Moderately expressing promoters such as the the *PEX8 *gene encoding a peroxisomal matrix protein that is essential for peroxisome biogenesis [90] and the *YPT1 *gene encoding a GTPase involved in secretion [91] have been described.

Recently the isocitrate lyase promoter (*PICL1*) has also described [20].

### PGAP

The glyceraldehide 3-phosphate dehydrogenase promoter (*PGAP*) has been used for constitutive expression of several heterologous proteins [17]. In *GAP *promoter expression system, the cloned heterologous protein will be expressed along with cell growth if the protein is not toxic for the cell. This system requires no washing to remove non-methanolic carbon sources, and no accurate optimization of the culture conditions as in methanol induction phase [92]. This system is more suitable for large-scale production because the hazard and cost associated with the storage and delivery of large volumes of methanol are eliminated [84]. These vectors allow for continuous production of the recombinant product avoiding the traditional *P. pastoris *fed-batch fermentations using the methanol inducible system. Thus, the features of the *GAP *expression system may contribute significantly to the development of cost-effective methods for large-scale production of heterologous recombinant proteins. [93,94].

In general, the substrates used with this promoter are glucose or glycerol. However, the constitutively expressed *P. pastoris PGAP *is strongly regulated by the carbon source. Döring and coworkers reported that the maximal transport activity of rPEPT2 growing on glucose was approximately 2 and 8 times higher than in cells grown on glycerol and methanol [95].

Some authors have reported that *GAP *promoter is more efficient that *AOX1 *promoter for heterologous protein expression. Delroisse and coworkers obtained two fold higher protein in shaking flasks (50 ml) batch fermentation [94]. Menéndez and coworkers showed that the enzyme production occurred three-fold more efficiently in the constitutive system than in the methanol-inducible one in fed-batch fermentation using glycerol as carbon source in the constitutive system [20]. Similar results were obtained with the *GAP *promoter growing on glucose in the bacterial β-lactamase production [17] and in a functional mammalian membrane transport protein production [95]. Although Goodrick and coworkers obtained similar expression level of a human chitinase using either *GAP *or *AOX1 *promoter under fed-batch fermentation, they developed a continuous strategy which provides not only for greatly enhanced production of recombinant proteins (five-fold higher productivity than fed-batch fermentation) and reduction of downtime associated with fermentation turnaround, but also for the production of intact proteins that are usually degraded in fed-batch fermentation [84].

However, other authors reported a higher production when using the *AOX1 *instead of the *GAP *promoter [92,96]. Nevertheless, it has been shown that increasing the copy number can enhance expression levels under *GAP *promoter in the production of HBsAg [96]. These authors have also implemented a culture bioprocess withdrawal and fresh medium replenishment through 20 cycles (semi-continuous culture) maintaining the production cycle to cycle and thus, increasing the total productivity of the bioprocess. Even some authors have reported the null protein production under *GAP *promoter due to the toxic effect of the protein on *Pichia *growth.

In order to increase the expression levels of *GAP *promoter the combination of *AOX1 *and *GAP *promoters to co-express recombinant proteins has been developed [93]. The idea is to induce *AOX1 *promoter with methanol after the exhaustion of glucose [93]. The secreted protein concentration was about two-fold higher compared to *GAP *promoter with appreciable differences in cell concentration obtained [93].

The combined use of *GAP *and *AOX1 *promoter in *P. pastoris *has been also developed to sequentially express and separately recover a constitutively intracellular β-galactosidase under *GAP *promoter and inductively hGM-CSF under *AOX1 *promoter and constitutively hGM-CSF and inductively hAS [97].

A resume of the results obtained with this promoter is presented in Table 6.

**Table 6:** *PGAP *promoter cultures comparison

**Protein**	**Promoter**		**Substrate**	**Total protein** **(mg/L)**^1 ^ **(U/mL)**^2^	**Productivity** **(mg/L h)**^1 ^ **(U/L h)**^2^	**Operational mode**	**Bioreactor**	**Reference**
Insect esterase	*GAP*	Intracellular	Glucose	7^1^	--	Batch	Shake flasks 50 mL	[94]
							
		Extracellular		80^1^	--			
				
	*AOX1*	Extracellular	Methanol	40^1^	--			

Fructose exo-levanase	*GAP*	Extracellular	Glycerol	26.6^2^	682^2^	Fed-batch	Bioreactor 7.5 L	[20]
			
	*AOX1*	Extracellular	Methanol	21.1^2^	220^2^	Fed-batch		

Human trysinogen	*GAP*	Extracellular	Glycerol	--	--	Fed-batch	Bioreactor 2 L	[21]
			
	*AOX1*	Extracellular	Methanol	--	--	Fed-batch		

Vitellogenin	*GAP*	Intracellular	Glucose	12^2^	--	Fed-batch	Bioreactor 2 L	[98]

hGM-CSF	*GAP*	Extracellular	Glucose	90^1^	1.2^1^	Batch	Shake flasks 50 mL	[93]
			
	*GAP+AOX1*	Extracellular	Methanol	180^1^	2.4^1^	Batch		

Aqualysin I	*GAP*	Extracellular	Glucose	1000^1^		Batch	Shake flasks	[100]

h-Chitinase	*GAP*	Extracellular	Glucose	450^1^	2.8^1^	Fed-batch	Bioreactor 3 L	[84]
						
				--	15^1^	Continuous		
			
	*AOX*	Extracellular	Methanol	350^1^	2.9^1^	Fed-batch		

HBsAg	*GAP*	Extracellular	Glucose	--	--	Fed-batch	Shake flasks 500 mL	[96]
					
	*GAP *multicopy	Extracellular		--	--	Fed-batch		
						
				--	--	Cyclic batch		
		
	*AOX1*	Extracellular	Methanol	--	--	Fed-batch		

Cellobiohydrolase	*GAP*	Extracellular	Glycerol	--	--	Fed-batch	Bioreactor 7.5 L	[92]
		
	*AOX1*	Extracellular	Methanol	--	--	Fed-batch		

Some authors have also pointed out that the secretion levels of protein compared with intracellular levels using *GAP *promoter are lower [94,98]. Some different genetic and physiological factors as proteolytic protein turnover [45], protein processing and folding in the endoplasmatic reticulum and Golgi apparatus [99], signal sequence [100] or secretion out of the cells have been pointed out as some of the causes to explain this fact [21].

Obviously, the target gene and the recombinant protein itself are key factors in determining the expression levels in *P. pastoris*. However, in some cases fermentation strategies are not optimized. Thus, the effect of the promoter on the levels of heterologous protein production obtained is very difficult to compare.

### PFLD1

Cloning and characterization of the formaldehyde dehydrogenase gene and promoter have been reported recently [18]. The *FLD1 *gene codes for an enzyme that plays an important role in the methanol catabolism as carbon source, as well as in the methylated amines metabolism as nitrogen source. In the methanol assimilation pathway [15], the first oxidation step takes place in the peroxisome, where the alcohol oxidase generates formaldehyde and hydrogen peroxide from methanol. The hydrogen peroxide is degraded by a catalase and a fraction of the formaldehyde is assimilated by the xylulose monophosphate cell biosynthetic pathway. The other portion leaved the peroxisome and it is oxidized in the cytosol via formate to carbon dioxide by a formaldehyde dehydrogenase and a formate dehydrogenase.

In addition to methanol metabolism, *FLD *is involved in the assimilation of some C_1_-amines such methylamine as nitrogen source [15]. The oxidation of methylamine takes place in the peroxisome by a methylamine oxidase, generating formaldehyde, hydrogen peroxide, and ammonium ions. This formaldehyde can, in a first step, be oxidized to carbon dioxide or assimilated to biomass following the same pathways involved in methanol metabolism. However, yeasts cannot use methylamine as a sole carbon and nitrogen source, and therefore, a supplementary source of easily metabolized carbon must be provided for sustained growth. The reason for this inability to exploit the methylamine carbon residue is unclear, but it is probably related to the observation that the methylamine oxidase synthesis is strongly repressed by ammonia [15]. The presence of an assimilation pathway of the methylamine carbon has been demonstrated in the yeast *Hansenula polymorpha *growing on glucose and methylamine [101] and the molecular characterization of the *Hansenula polymorpha FLD1 *gene encoding formaldehyde dehydrogenase has been reported [102].

Initial characterization studies showed that the *PFLD1 *from *P. pastoris *was strongly and independently induced either by methanol as carbon source or methylamine as nitrogen source [18]. The gene *FLD1 *has been used also as a novel selectable marker for DNA-mediated transformations of *P. pastoris *[103]. However, the selection of optimal operational strategies for heterologous protein production under this promoter is in an incipient stage.

Batch cultivation experiments expressing a *Rhizopus oryzae *lipase gene under the transcriptional control of the *PFLD *were made using sorbitol and methylamine as carbon and nitrogen sources [29]. These results strongly indicated that sorbitol, a carbon source that does not repress the synthesis of methanol metabolism enzymes [73], also allows the induction of *PFLD *by methylamine. This suggested that the use of a sorbitol as carbon source combined with methylamine as nitrogen source could be the basis for the development of methanol-free fed-batch fermentation processes for heterologous protein production in *P. pastoris *based on the *PFLD*. A simple and reliable fed-batch strategy has been recently developed using the *PFLD *[104]. The operational strategy consists in three phases. A GBP using glycerol and ammonium as carbon and nitrogen source respectively in stoichiometric relation to achieve the exhaustion of both substrates at the end of the GBP. A second sorbitol-methylamine batch phase (SMBP) as a transition phase. This phase allows pre-adapting *P. pastoris *cells metabolism to the carbon and nitrogen sources used in the induction phase. Third, a methylamine induction phase (MAIP) when in a first attempt sorbitol levels were near exhaustion by means of a pre-programmed exponential feeding rate strategy ensured a constant specific growth rate along the MAIP. Different fed-batch made at different specific growth rates showed the important effect on secreted recombinant protein productivities of this parameter. Results suggested that extracellular ROL production seemed to be favoured when the microorganism was growing at higher growth rates and, therefore, in a situation of carbon excess. A new fed-batch strategy where the feeding rate was manually programmed to maintain sorbitol in excess of 8 g l^-1 ^was performed. The results obtained in terms of maximal lipase activity, lipase yield and productivities increased at least two fold compared to pre-programmed exponential feeding rate strategy. The authors demonstrated that *PFLD *system can be successfully used for protein production in *P. pastoris *using methanol -free high cell density cultivation strategies, being comparable in terms of process productivity to the *PAOX*-based system in terms of process productivity [104].

### PICL1

Recently, a gene encoding isocitrate lyase from the methylotrophic yeast *P. pastoris *has been cloned and characterized. Expression of the dextranase gene (dexA) from *Penicillium minioluteum *under control of *ICL1 *promoter was regulated in response to the carbon source, being the expression of the protein controlled by the culture conditions [20]. Thus, *PICL1 *is a good alternative for the expression of heterologous protein in the methylotrophic yeast. However no bioreactor experiments have been reported and further studies must be necessary to optimize the promoter and the fermentation conditions.
